# A New Metre for Cheap, Quick, Reliable and Simple Thermal Transmittance (*U-Value*) Measurements in Buildings

**DOI:** 10.3390/s17092017

**Published:** 2017-09-03

**Authors:** José Manuel Andújar Márquez, Miguel Ángel Martínez Bohórquez, Sergio Gómez Melgar

**Affiliations:** Escuela Técnica Superior de Ingeniería, Universidad de Huelva, Ctra. Palos de la Ftra.-Huelva s/n, 21819 Huelva, Spain; andujar@uhu.es (J.M.A.M.); sergio.gomez@dimme.uhu.es (S.G.M.)

**Keywords:** *U-value*, thermal transmittance, energy retrofitting of existing buildings, wireless, heat flow, temperature sensors, Raspberry Pi, Zigbee, Xbee, Arduino

## Abstract

This paper deals with the thermal transmittance measurement focused on buildings and specifically in building energy retrofitting. Today, if many thermal transmittance measurements in a short time are needed, the current devices, based on the measurement of the heat flow through the wall, cannot carry out them, except if a great amount of devices are used at once along with intensive and tedious post-processing and analysis work. In this paper, from well-known physical laws, authors develop a methodology based on three temperatures measurements, which is implemented by a novel thermal transmittance metre. The paper shows its development step by step. As a result the developed device is modular, scalable, and fully wireless; it is capable of taking as many measurements at once as user needs. The developed system is compared working together on a same test to the currently used one based on heat flow. The results show that the developed metre allows carrying out thermal transmittance measurements in buildings in a cheap, quick, reliable and simple way.

## 1. Introduction

The reduction of energy demand is the easiest way to limit the production of greenhouse gases. It can be made possible through behavioural changes or higher energy efficiency. The energy demand of buildings (houses, public and private offices, shops and other buildings) represents a very important part of the European Union’s (EU) final energy consumption [1,2,3], nearly 40%; and it is expected to continue to grow in the coming years. By 2030 the EU will depend on 90% of oil imports and 80% on natural gas to cover its energy needs. Improving the energy efficiency of buildings, responsible for one-third of greenhouse gas emissions, would not only save costs but also partially offset the impact of climate change [4]. The EU’s Horizon 2020 programme emphasises the need to reduce the difference between calculated and actual performance of buildings by increasing their energy efficiency [5]. Usually, the energy consumption in buildings is conditioned by the use and maintenance of their facilities as well as their constructive characteristics (mainly insulation).

Human thermal comfort depends on many factors including personal (physiological, sociocultural, etc.) and environmental. To achieve a certain level of thermal comfort two sets of actions can be carried out: passively (building envelope) and actively (heating and cooling systems). 

While it is true that in the developed countries new buildings are subject to strict constructive norms to achieve high energy efficiency; it is also true that, in these countries and in general, there is a huge building park that was built with less demanding criteria (and depending on the lifetime, perhaps with none). This huge body of existing buildings needs to be energy retrofitted to achieve levels of efficiency that align with current requirements.

It is estimated that if a good building energy retrofitting is carried out up to 27% of energy saving can be achieved [3]. Based on our own experience the result can be even better depending of course on the initial state of the building and the extent and quality of retrofitting achieved.

In the context of building energy retrofitting, the measurement, processing and interpretation of the variables of interest play an important role. Usually this is done with specific data acquisition systems (DAQ). They provide the retrofitting measures for the variables of interest both before (to assess the retrofitting to be carried out) and after (to evaluate the retrofitting results and obtain the reliability of improvement). In general, measurements of temperature [6,7,8,9] and relative moisture [10,11] both indoor and outdoor, solar radiation [12,13], envelope thermal transmittance (*U-Value*) [14,15], power consumption by electrical circuits [16], etc., are required.

This work is devoted to the measurement of one of the above-cited variables, perhaps the most complex to measure, the *U-Value*. For this, a novel wireless measurement system has been developed, which provides simple, quick and cheap measurement of *U-Values* in buildings.

The better-insulated a structure is, the lower the *U-Value* will be. The *U-Value* (reciprocal of thermal resistance, *R_t_ value*) is the rate of transfer of heat through a structure, which can be a single material or a composite, divided by the difference in temperature across that structure. The units of measurement are W/(m^2^ K). Currently, the three most common methods for measuring the *U-Value* in buildings, although there are others [17,18], are:

(a) Theoretical calculation using the ISO 6946: 2007 standard [19,20,21]. The procedure may be invasive (making coves in the building envelope) or not, and it is necessary to take into account the thickness of each of the layers that compose the building envelope as well as its thermal conductivity, which is calculated according to ISO 10456: 2007 standard [22]. This method is of approximation, and the obtained *U-Value* is often far from the actual [23,24,25]. This is mainly due to the fact that in existing buildings, theoretical evaluation of *U-Value* can be difficult because the materials used in the envelopes are not usually homogeneous and their values are unknown, and also because the necessary application of destructive methods is not always feasible.
(b) Based on the direct measurement of the heat flow through the building envelope according to ISO 9869: 2014 standard [26]. For this, a flow heat metre and a pair of temperature sensors [27] are used. The *U-Value* measurement must be done once the steady-state has been reached, i.e., the average heat flow on both envelope sides must be equal during a long-enough period of measurement. However, from a physical-mathematical standpoint, steady-state conditions are never achieved; therefore, in practice, we can assume that average values of heat flow rate over a reasonably long period of time (minimum 72 h) give an estimate of the steady-state conditions.
(c) Measured through the infrared thermovision technique, which consists of calculating the *U-Value* through thermographic camera measurements and the subsequent analysis of the images obtained [28,29,30]. Although (a) it is also a very imprecise method, it is a very interesting approach for taking a first look.

The most widely accepted of the above three methods for its accuracy is (b). However, measuring the *U-Value* becomes very tedious, time consuming and expensive for the following reasons:

1. The duration of the test must exceed 3 days taking up to 7 to 14 days or even more than a month for heavy structures; or over a year in the case of a ground floor slab, due to heat storage in the ground.
2. If we try to apply this method in building energy retrofitting and, as usual, the number of measures to be carried out is high, its application is not feasible. We would need months or years to carry out the measurements, or a large number of equipment measuring at the same time, which would be impractical for its price and processing costs.
3. The accuracy of measurements depends on a number of factors:
  ○ Magnitude of temperature difference (larger = more accurate).
  ○ Weather conditions (cloudy is better than sunny).
  ○ Good adhesion of the head flow sensor to test area.
  ○ Duration of monitoring (longer duration enables a more accurate average).
  ○ More test points enable greater accuracy to mitigate anomalies.
4. The heat flow metre is a relatively expensive and unstable device, as well as needs a specific instrumentation channel. The total cost of sensor + instrumentation + processing + data display may be several thousand of € for each measurement point.

The system that has been developed in this work aims at overcoming all the above-cited limitations by providing an easy, quick and cheap *U-Value* measurement. The measurement methodology used is derived from (b); it consists of the *U-Value* direct measurement without using a flow heat metre. Specifically, the measurement of three temperatures is only required: Environment on each face of the structure and surface on one of them. The temperature sensors used have been patented by the authors of this paper [31,32], although others may be used.

Regarding the duration of the test, according to method (b), all the mentioned time periods (3 days taking up to 7 to 14 days or even more than a month for heavy structures; or over a year in the case of a ground floor slab) are estimated from a conservative point of view: in the sense that experience advises these time periods to ensure the reliability of the measure in any case. However, based on our own experience, the most important factor for a good measure of the *U-Value* is not so much the time during which it is being measured, but the time it takes the measurement to stabilize (to reach its steady-state value). Actually the casuistry in the measure is very variable depending mainly on the thermal gradient interior/exterior and the nature of the structure. Therefore it would be very relevant to have a system that is able at all times, based on the above, to decide when a measure is correct (it has reached its steady-state) and when not. This would allow fitting the measurement time in each case, opening the possibility of taking more measures in less time and therefore generating a significant saving. This is precisely another of the most important features of our developed system: if during a user-adjustable time the measurement remains in its steady-state value within a threshold also user-adjustable, the system can automatically take the measurement (if it is programmed for that) and ends the test automatically as well. This is possible because the developed system works online and processes data in real time; the known commercial systems work offline as data loggers. 

The developed *U-Value* metre allows for taking multiple measures at the same time, which also allows for considerably cutting the time needed to have a suitable set of measures in a retrofitting of an existing building. In fact, it is fully modular and configurable by the user according to his/her needs.

The developed *U-Value* metre incorporates a user interface (implemented by a virtual instrument, VI) for its remote handling. It receives real time measurements, processes and stores them. The VI allows detecting communication/sensor errors and failures, and warns of anomalies. Finally, the user can create graphs and reports by using the VI. Once the temperature sensors are placed, no process of obtaining the measurements requires human intervention.

Finally, in order to have experimentation results, the developed system is used to measure the *U-Value* in a dwelling that has undergone energy retrofitting. Moreover, taking advantage of the mounted experimentation, the developed system is compared, working together on a same test, to the one currently used based on heat flow measurement. The results show that the developed metre allows carrying out thermal transmittance measures in buildings in a cheap, quick, simple and reliable way. 

The rest of the paper is organized as follows: in Section 2 that follows the measurement methodology is presented, this is the mathematical foundation of the developed system. Section 3 is devoted to analyze the developed *U-Value* metre, both the hardware and software. In Section 4 the way to carry out measurements and the results thereof is shown; it also includes a comparison with the currently used technology. In discussion, Section 5 the main features and strengths of the developed *U-Value* metre with respect to the current technology are highlighted. Finally, Section 6 outlines the main conclusions of the paper. 

## 2. Measurement Methodology

**Figure 1:**
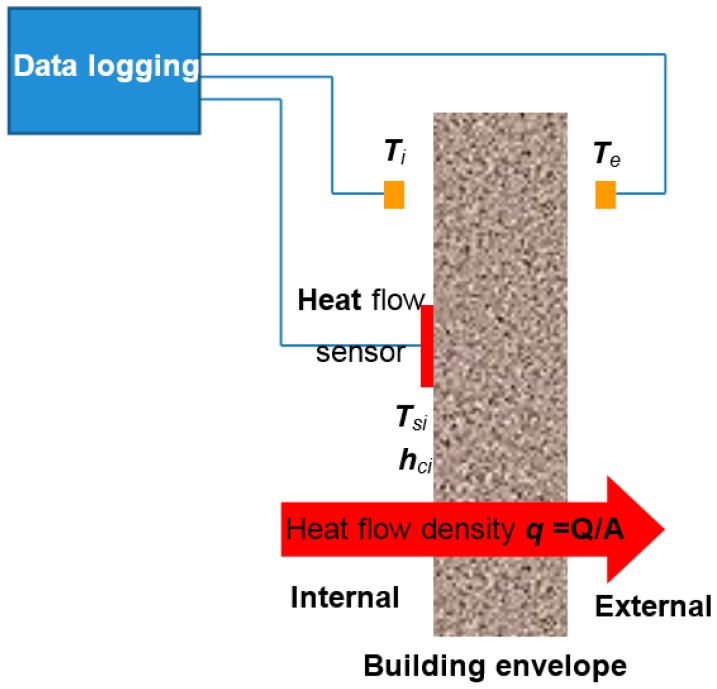
In situ *U-Value* measurement according to ISO 9869: 2014 standard (average method); it is assumed that $T_{i}>T_{e}.$

*U-Value* measures show how effective a material is as an insulator. Assuming a mono-dimensional heat flow and steady-state conditions (see Figure 1), the *U-Value* of a system is defined as the heat flow rate per unit area, *q*, divided by the temperature difference between the surroundings on each side of the system. This is:

(1) $$U-Value=\frac{q}{T_{i}-T_{e}}$$

where, if as usual, the system is a wall with an indoor side and an outdoor one. *T_i_* and *T_e_* are, respectively, the indoor and outdoor surrounding temperatures. As *q* is always flowing from the hottest temperature to the coldest, the practical application of Equation (1) requires to take into account if $T_{i}>T_{e}$ or not, since this sets the direction of *q* (leaving the wall or entering it, respectively). In order to avoid negative *U-Values*, it is enough to consider the absolute value $\left|T_{i}-T_{e}\right|,$ or simply change the order of the temperatures in the parentheses; in this way you can perform bidirectional heat flow measurements (both in winter and summer) from the indoor of the wall. 

From the point of view of building construction, the *U-Value*, assuming steady-state conditions, is the amount of heat flowing per unit of surface through a specific building envelope when there is a temperature gradient between its indoor and outdoor surrounding.

**Remark.**  Steady-state condition does not mean that the U-Value reaches a constant final value, which is impossible according to (1) due to the continuous temperature changes. The meaning is that the average U-value remains substantially constant over time.

Following Equation (1), the ISO 9869: 2014 [26] standard for measuring the *U-Value* (see Figure 1) requires two temperatures sensors and one heat flow metre on the side with the most stable temperature, usually indoor. The heat flow sensor (thermopile) should be in direct thermal contact with the surface of the envelope (usually a wall) over the whole area of the sensor. Regarding the temperature sensors, they must measure the surrounding air temperature (not the envelope surface temperature). 

The steady-state assumption implies that only one heat flow metre is enough, since the average heat flow on both sides are equal in a long-enough period of measurement. However, how long does a long-enough period of measurement last? It depends on the type of test carried out. A conservative way is to wait long enough (given by our experience and set in hours or days in the standards) and apply it, as caution, to all tests of the same type. Another more interesting way from the research point of view is to be able to detect when the steady-state is reached to end automatically the test. This is one of the key issues of this paper.

Instant measurements (*q*, *T_i_* and *T_e_*) are processed by using the progressive average procedure. It is based on the idea that the average of instantaneous ratios between heat flow and temperature differences on a progressively increasing time scale smoothes out oscillations leading to the steady-state value of the thermal transmittance. The equation that describes the procedure is:

(2) $$U-Value\;\left(\frac{W}{m^{2}K}\right)=\frac{\sum_{j=1}^{n}q_{\left(j\right)}}{\sum_{j=1}^{n}\left(T_{i\left(j\right)}-T_{e\;\left(j\right)}\right)}$$

where *n* is the number of measures made during the test. 

Equation (2) delivers a *U-Value* close to the real if the following conditions are met [26]:

1. The steady-state is achieved.
2. The heat-flow sensor is not exposed to direct solar radiation; it is better to always measure from the inside.
3. The temperature difference between indoor and outdoor must be greater than 10 °C.
4. The wind speed must be less than 3 m/s.
5. The thermal conductivity of the all involved elements is constant during the test.

In order to find a more practical way of measuring the *U-Value*, we can use a methodology based on the Newton’s law of cooling. The law states that when the temperature difference between a body and its surrounding is not too great, the rate of heat transferred (to or from the body) by conduction, convection and radiation is practically proportional to the temperature difference between the body and the surrounding. This is:

(3) $$\frac{dQ}{dt}=\alpha \;S\left(T-T_{s}\right)$$

where:

*Q* is the thermal energy in joules.
α is the heat transfer coefficient (W/(m^2^ K)).
*S* is the heat transfer surface area of the body (m^2^).
*T* is the body temperature.
*T_s_* is the surrounding body temperature.

Applying Equation (3) to Figure 1 with the condition that the heat transfer surface area of the body is the wall, we can write that

(4) $$\frac{dQ}{dt}=h_{ci}S\left(T_{i}-T_{si}\right)$$

where *h_ci_* is the *indoor surface convective heat transfer coefficient* (W/(m^2^ K) of the wall, *T_si_* its *indoor surface temperature*, *T_i_* its *indoor surrounding temperature*, and considering that the building is losing heat through its envelope. This is, the fluid (air surrounding the inner wall) extracts heat from the environment and changes its density causing it to move to the cooler side (outer wall) where it delivers its heat. If the *q* flow is opposite to that of Figure 1 (typical situation in summer, over all in subtropical countries), the inner wall delivers heat to its surrounding air, thus the parenthesis in Equation (4) must be rewritten as $T_{si}-T_{s}.$

According to Figure 1 and taking into account Equations (1) and (4) in steady-state, we can write for a building envelope that

(5) $$q=U-Value\left(T_{i}-T_{e}\right)=h_{ci}\left(T_{i}-T_{si}\right).$$

From this equation the *U-value* can be obtained on the basis of only three temperature measures, i.e.,

(6) $$U-Value=h_{ci}\frac{T_{i}-T_{si}}{T_{i}-T_{e}}\cdot$$

Finally, by following the progressive average procedure to lead to the steady-state value of the thermal transmittance,

(7) $$U-Value\left(\frac{W}{m^{2}K}\right)=h_{ci}\frac{\sum_{j=1}^{n}\left(T_{i\left(j\right)}-T_{si\left(j\right)}\right)}{\sum_{j=1}^{n}\left(T_{i\left(j\right)}-T_{e\left(j\right)}\right)}\cdot$$

Equation (7) allows obtaining the *U-Value* of a building envelope by measuring three temperatures (see Figure 1), namely: *T_i_*, *T_e_* and *T_si_.* The *h_ci_* values can be obtained from the standard [19] (page 13), i.e., *h_ci_* = 5.0 (W/m^2^ K) for heat flow upwards; *h_ci_* = 2.5 (W/m^2^ K) for heat flow horizontal and *h_ci_* = 0.7 (W/m^2^ K) for heat flow downwards. Regarding these *h_ci_* values there is some dispersion depending on the used bibliography, for that, it is best to use the standard. In any case and regarding our methodology this is not a problem because we make differential measurements, i.e., we measure before and after the building energy retrofitting, and then we subtract the measurements in order to get the obtained improvement.

In practice, in order to minimize errors in the envelope surface temperature measurement, it is advisable to carry out several measuring at the same time around the measurement zone. Specifically and based on our own experience, it is a good practice to take three measurements in points spaced about 10–15 cm from each other. In this way, the chosen *T_si_* to introduce every *j* in (7) will be the average of these three measurements. Of course, if there is a lot deviation of one of the measurements, it must be discarded, as it is probably due to malfunctioning of its respective sensor.

## 3. Developed *U-Value* Metre

**Figure 2:**
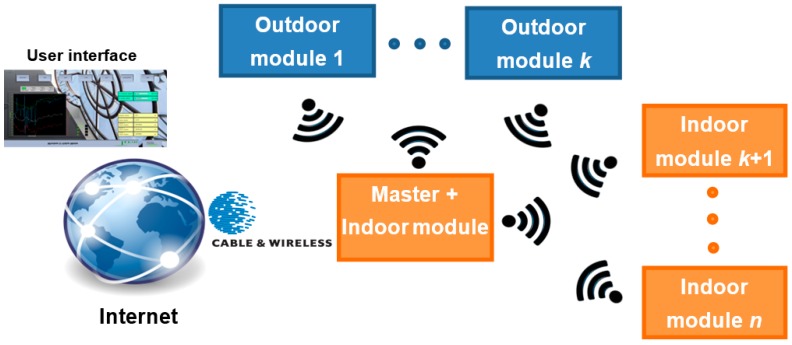
Developed *U-Value* metre.

The *U-Value* metre design is fully modular and also its use. It is made up of different modules that the user can configure according to his/her need (Figure 2). The indoor and outdoor modules are physically separated but connected via wireless. All modules function as *Xbee* devices using *Zigbee* protocol [33,34].

*Xbee* is a small (about 2 cm^2^), low-power (tens of mW) digital radio. Since its initial introduction (2005), a number of new *XBee* radios have been developed and an ecosystem of wireless modules, gateways, adapters and software has evolved. Today, through an *Xbee* device, any small single-board computer can be integrated in any specific purposeful wireless network.

Regarding *ZigBee*, it is an IEEE 802.15.4-based specification [35,36] used to create personal area networks. The technology defined by the *ZigBee* specification is intended to be simpler and less expensive than other wireless personal area networks such as bluetooth or wi-fi.

Depending on the *XBee* device used, the combination *XBee* + *ZigBee* allows a communication range of more than 100 m in closed spaces and 1 km in open spaces. In any case, *ZigBee* devices can transmit data over long distances by passing data through a mesh network of intermediate devices to reach more distant ones. This feature is implemented in our developed *U-Value* metre.

Taking into account that a *ZigBee* network can consist of a maximum of 65,535 nodes distributed in subnets of 255 nodes, the ability of the developed *U-Value* metre, according to the configuration of Figure 2, is practically unlimited, and certainly higher than any practical requirements infield.

If on a building only one *U-Value* measurement is needed to be taken, the configuration of the developed *U-Value* metre is the simplest: the *master + indoor* module and one *outdoor* module. Following Figure 1, the *master + indoor* module takes the temperatures *T_i_* and *T_si_* (three measurement points) and the outdoor module takes the temperature *T_e_*. The *master + indoor* module collects all the measures, processes them following Equation (7) (it can also make partial *U-Value* calculations by Equation (7) if so programmed from the user interface) and saves (in an internal memory) the calculated *U-Value*. At the same time the metre can transmit all measures in real time internet connection (even using mobile phone network or a modem radio) via cable, Wi-Fi or wireless. In our case, we can receive in our laboratory all the real time measures from any building in any part of the world. For that we have developed a user interface (by a *virtual instrument*, VI) as part of the developed *U-Value* metre. Of course, data can be also stored in situ, and with a USB memory device in the *master + indoor* module. From this initial and simple configuration, the user can scale the developed *U-Value* metre according to his/her needs, i.e., adding as many *indoor* and *outdoor* modules as needed. Taking into account the distance range and capabilities of incorporating modules, entire blocks of buildings with the same *U-Value* metre can be monitored simultaneously. In addition, what is more important still, all measures can be done at once and their values shown in real time.

### 3.1. Master + Indoor Module

**Figure 3:**
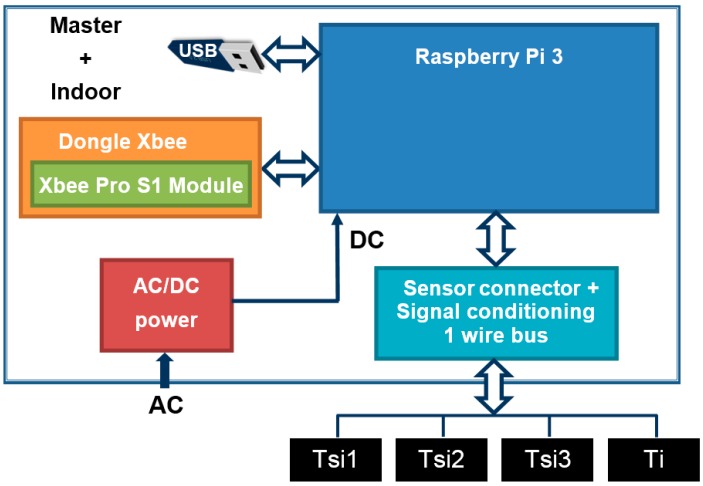
*Master + indoor module* block diagram.

The block diagram of the *Master + indoor module* is shown in Figure 3. It is based on a small single-board computer *Raspberry Pi 3* [13,37]. In addition to the *Raspberry Pi 3*, the *master + indoor* module has a built-in *Dongle Xbee* + *XBee* device (*Xbee S1 Pro*, [38]), an AC/DC power supply, and a board were the temperature sensors are connected. It allows connecting the 1-wire bus used with the temperature sensors (for measuring *T_i_*, and 3 *T_si_*) to the *Raspberry*. Finally, although the measures can be transmitted in real time to any point with Internet connection, these are also stored in an external USB memory device (32 GB in this prototype), which allows for measure recovery if any communication failure occurs. This USB memory device can be removed by the user.

As for functioning, the *master + indoor module* sends a synchronizing signal with information of the measure time interval to all the modules involved at the beginning of the test; from here each module can operate independently. This avoids losing measures in case of wireless communication link failures. If a module cannot be synchronized, the user receives a beep signal, and warning is displayed on the user interface.

Regarding Equation (7), the *master + indoor module* takes four temperature measurements (*T_i_* and 3 *T_si_*) for an adjustable time interval by the user, or automatically until the *U-Value* reaches the steady-state. This can be done from the user interface. For calculating the average value of the 3 *T_si_*, the *master + indoor module* checks first the deviation among the three measures; if any of them exceeds a fixed threshold (set by the user) this measure is discard and the user receives a beep that is also displayed on the user interface. The probable cause is that a temperature sensor is malfunctioning or perhaps badly fixed to the wall.

As an example of *U-value* test, if its duration is 3 days and the measures are taken each half hour, *n* in Equation (7) is 72/0.5 = 144, so 144 measures of *T_i_* and *T_si_* (average) are taken. The 144 measures are recorded in 2 columns (*T_i_* and *T_si_*). There is another that contains a reference of the time *t* when measures were taken. In addition, this module also receives from the outdoor module the 144 *T_e_* measurements, taken at the same time that *T_i_* and *T_si_*. At the end of the test, the data storage comprises a matrix of 4 columns (*t*, *T_i_*, *T_si_* and *T_e_*) and 144 rows. With these data and applying Equation (7), the *U-Value* is calculated and shown on the user interface. Of course, if the module has been programmed in this way, at any time of the test or even continuously, partial *U-Value* calculations can be carried out.

**Figure 4:**
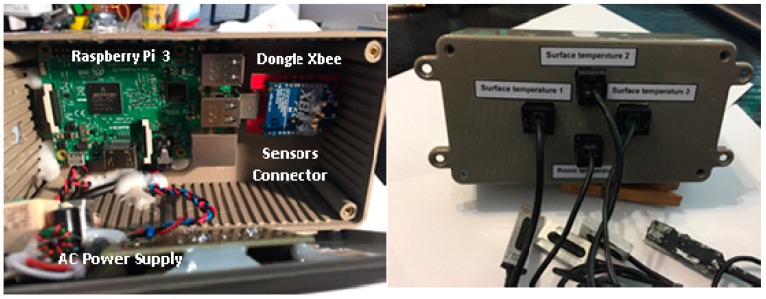
*Master + indoor module* prototype: Internal (**left**) and external (**right**) views, with its four temperature sensors.

Figure 4 illustrates the actual built module. The *master + indoor* module is housed in a plastic box which can be plugged directly into a 230 V AC electrical wall outlet and operates in plug and play mode. The cost of this prototype, temperature sensors including, is around 150 €. This price only takes into account the cost of materials; in terms of labor has been carried out by hand. Of course, for industrial production the costs would be greatly reduced.

### 3.2. Indoor Module

**Figure 5:**
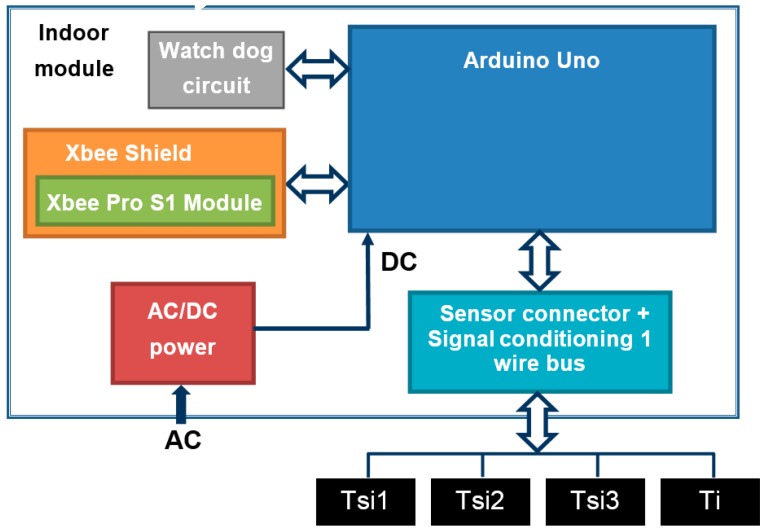
*Indoor module* block diagram.

The block diagram of this module is shown in Figure 5. It is based on a microcontroller board *Arduino Uno* [39,40,41]. Because this module needs less computing requirements than the *master + indoor module* (since it will always act as its slave), we have decided to use an *Arduino Uno* instead of a *Raspberry Pi 3*. The fundamental difference between both is that *Raspberry Pi* is a fully functional computer, whereas *Arduino* is essentially a microcontroller. 

In addition to the *Arduino Uno*, the *indoor* module has built-in a *Shield Xbee* + *XBee* device (*Xbee S1 Pro*), a *watch-dog circuit* (automatically resets the module in the event of communication blocks, which occurs with certain frequency in these devices), an AC/DC power supply, and a board where the temperature sensors are connected. It allows connecting the 1-wire bus used with the temperature sensors (for measuring *T_i_*, and 3 *T_si_*) to the *Arduino Uno*. 

Following the same *U-value* test example in Section 3.1, this module sends each temperature measurement (*T_i_* and *T_si_* average) referenced in time to the *master + indoor module*, but stores them as well. This allows you to recover the measurements if the wireless communications link fails.

**Figure 6:**
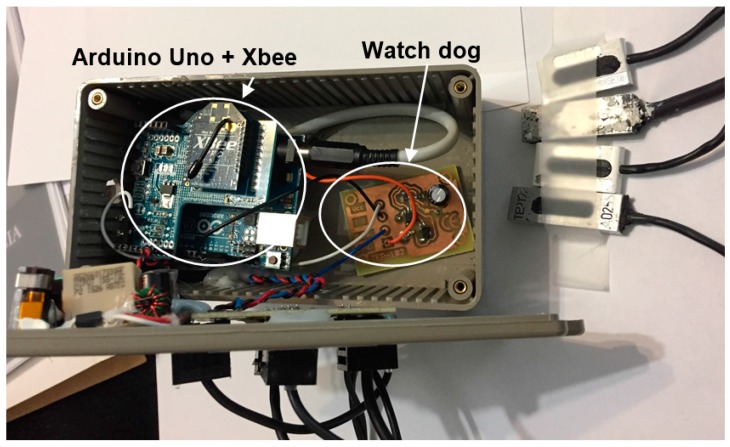
*Indoor module* prototype with its four temperature sensors (top right).

Figure 6 shows the actual built module. The *indoor* module is also housed in a plastic box which can be plugged directly into a 230 V AC electrical wall outlet and operates in plug and play mode. The cost of this prototype, sensors included, is around 90 €. As in the previous module, this price only takes into account the cost of materials.

### 3.3. Outdoor Module

**Figure 7:**
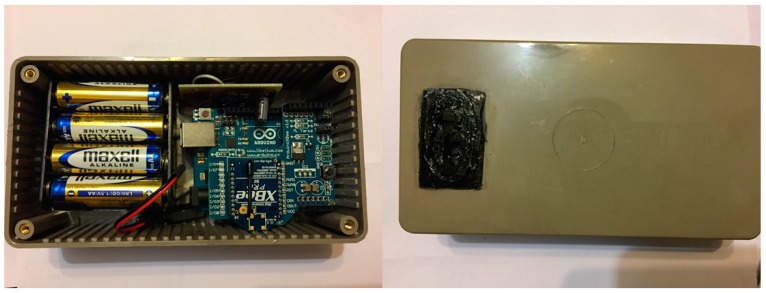
*Outdoor module* prototype (**left**) and its weather protected temperature sensor (**right**).

The *outdoor module* is essentially analogous to *indoor module* (Figure 5) with the exception that it has a single temperature sensor (*T_e_*) integrated in the box itself, which is weather protected; and it uses its own battery power source (Figure 7).

Following the same *U-value* test example in Section 3.1, this module sends each measure (*T_e_*) referenced in time to the *master + indoor module*, but it stores them as well. This allows for recovering the measurements if the wireless communication links fail.

The cost of this prototype, sensors and batteries included, is around 85 €. Again, this price only takes into account the cost of materials.

### 3.4. User Interface

The user interface was developed through a virtual instrument (VI) created in LabVIEW^TM^. This VI receives the real time measurements, processes and stores them. The developed VI allows detecting communication/sensor errors and failures, and warns of anomalies. By way of the VI the user can produce graphs and reports.

The VI is usually hosted in a PC; in our experimentation case (please see Section 4) we used a laptop. With the VI, the user can set all the features of the developed *U-Value* metre. Among them, the ability to perform a test in programmed time or by means of the continuous *U-Value* calculation until reaching the steady-state value.

**Figure 8:**
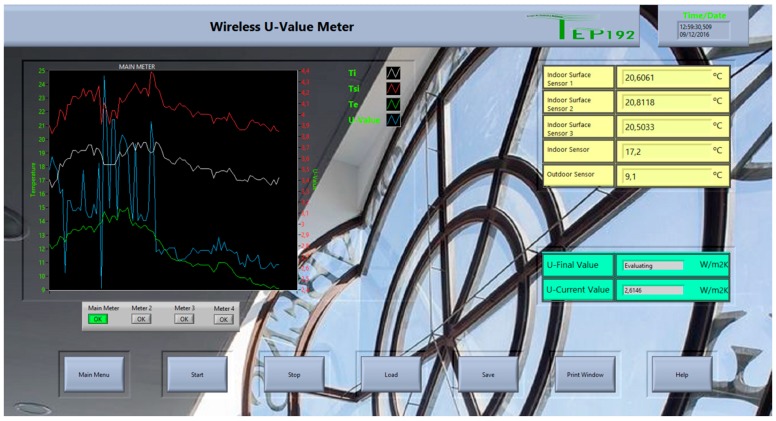
User interface front panel.

Figure 8 shows the front panel of the developed VI taking measures. It is user-friendly, very intuitive and simple, and it notifies the user when the *U-Value* is available or not. Measurements are carried out automatically and without user intervention.

## 4. Experimentation and Results

Initially and as specified in standard [26], a preliminary inspection of the wall to be measured with the thermographic camera must be carried out. The reason for this is to avoid points where there could be thermal anomalies such as a thermal bridge.

**Figure 9:**
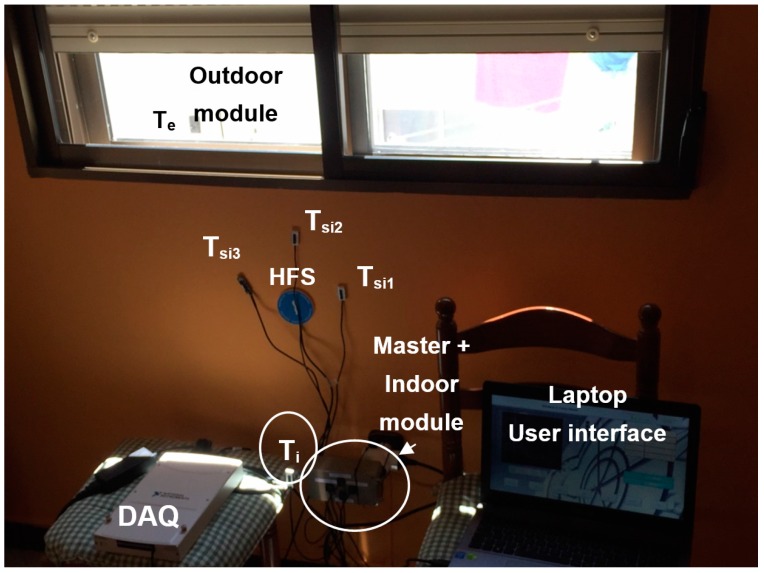
*U-Value* measurement experimentation assembly.

In order to compare both *U-Value* measurement methods, by direct heat flow measurement (2) and by three temperatures measurement (7), we have configured the experimentation scenario shown in Figure 9. The heat flow sensor (HFS) is the Hukseflux^TM^ mod. HFP01-05. This sensor can carry out bidirectional heat flow measurements with a sensitivity of $60\times 10^{-6}$ V/(W m^2^). The heat flow metre has been configured by the National Instrument ^TM^ DAQ mod. NI USB 6281. The DAQ is connected to a laptop (which hosts the VI) using a USB port. This allows synchronizing both experiments and processing their data.

As shown in Figure 9, the three surface temperature sensors (the separation between them is about 10–15 cm) are adhered by heat-conductive adhesive to the wall to be measured. This allows for taking an average measure $\left(T_{si}=\left(T_{si1}+T_{si2}+T_{si3}\right)/3\right)$ that is closer to the actual wall. *T_si_* is automatically calculated by the *master + indoor* module. Regarding the indoor temperature sensor (*T_i_*), which is also used for the heat flow metre, it is placed 30 or 40 cm from the wall at the height of the others (in closed and inhabited rooms, there is usually a thermal gradient between the floor and the ceiling, whereby the ambient temperature is not the same at different heights). 

The *outdoor module* is placed on the other side of the wall; in this case out of the dwelling. As we have already explained, its function is to take the external environment temperature *T_e_*, which is also used for the heat flow metre. It can be placed in the most comfortable place taking into account that the measured temperature will be the same at different heights (of course within small distances and avoiding sun and shadow changes). All temperature sensors used have digital output in 1-Wire format [42].

Once all temperature sensors are placed, the user just needs to plug the *master + indoor* module into the electric current and measurements automatically start (in our specific experimentation we also needed to synchronise both experiments, which was carried out through the laptop). The developed *U-Value* metre transmits the data, and at the same time processes and save them into its USB memory device.

In both experiments, in order for the *U-Value* to be valid, it must be measured according to standard ISO 9869-1:2014 [26]: the temperature difference between indoor and outdoor must be greater than 10 °C, the wind speed less than 3 m/s and the solar radiation received must not be direct.

In the developed *U-value* metre case, the described assembly must be repeated for each measurement point, but only using the *indoor* and *outdoor* modules; the user just programmes all the functions in the VI. In contrast, with the heat flow metre, the user needs as many full instrumentation channels as measurement points.

### Results

The experimentation was carried out in a dwelling, which underwent an energy retrofitting action. Before retrofitting, *U-Value* measures were taken using only the developed system. Certainly we would have wanted to carry out the complete assembly of the experiment (both methods as in Figure 9), but the immediate beginning of the action prevented it. In any case we were going to have time to compare both methods afterwards, upon energy retrofitting conclusion. Actually that is what we did. 

**Figure 10:**
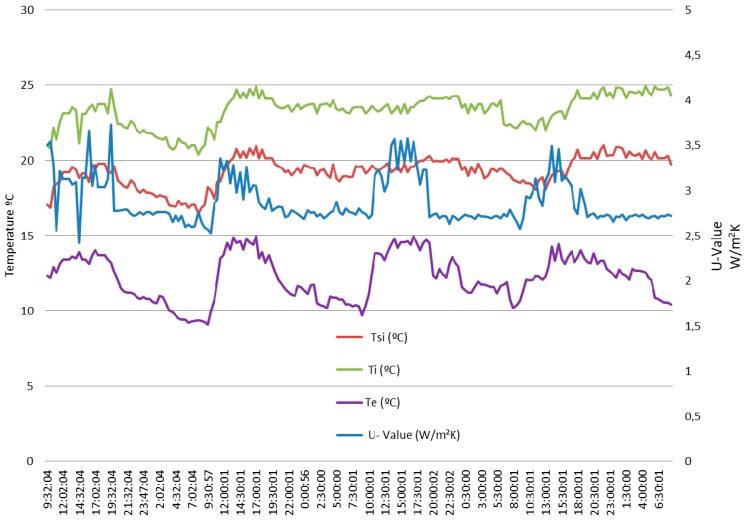
Measures taken with the developed *U-Value* metre before the energy retrofitting.

Figure 10 shows the measures in the dwelling before its energy retrofitting. At a first glance, the test was carried out in winter, this is, $T_{i}>T_{e}.$

The duration of the test was practically 4 days with measurements taken every half hour. A total of 188 measures were taken, whereby *n* in Equation (7) is 188. At the end of the test, the calculated *U-Value* by Equation (7) was 2.50 W/(m^2^ K). 

The left axis of Figure 10 is scaled in Celsius degrees, and the right in W/(m^2^ K), while the horizontal axis is scaled in time. The VI sets automatically the scale and points for better representation; therefore, in general, the timescale will not match with the measurement time. If the user needs individual sampling measurement values, the VI data matrix (188 rows and 4 columns, 752 points in this case) can be conveyed in spreadsheet format.

At this point, it is interesting to point out a very important feature of the developed system. As already established in Section 2, steady-state condition (necessary for measures to be valid) means that the average *U-Value* remains substantially constant over time. For this reason and as a precaution, the standard establishes a measurement time of at least 3 days, however in some cases this time may be insufficient. Where is the key then? Precisely in knowing whether the steady-state has been reached or not. If it has been reached the test can stop, if not the test must continue until it is reached. This way of facing the problem is novel and from our point of view very interesting, since it supposes the duration of test that in general will be different for each case; what is the duration then? It is strictly the necessary.

As the developed system performs in real time, during the test it is possible to apply Equation (7) as many times as it is programmed by the user. In this way, the obtained *U-Value* at any time of the test can be compared with the one obtained in the previous 12 or 24 h (or another period of time also programmable). If the deviation is less than a percentage (also programmable), it can be considered that the steady-state has been reached and therefore the test can stop. As an example, by taking the values of the first 24 h of the data shown in Figure 10, the obtained *U-Value* is 2.61 W/(m^2^ K). If a window of +/−5% is set, *U-Values* among 2.48 and 2.74 would be valid. This means that the test would not have needed four days, since the value calculated at the end has been 2.50 W/(m^2^ K). Of course this was just an example and the result will surely be different in another test, but the important thing is to have the ability to adjust dynamically and automatically test duration to the conditions in each case.

Once the retrofitting process has completed, we configured the experimentation scenario shown in Figure 9. This involved two tasks: (a) measuring *U-Value* improvement, and (b) comparing both *U-Value* measure methods, i.e., through the direct heat flow measurement (2) and through the three temperatures measurement with the developed metre Equation (7). For carrying out this experiment it was necessary to synchronise both metres, which was carried out in the laptop; in this way all temperatures were the same for both metres. 

**Figure 11:**
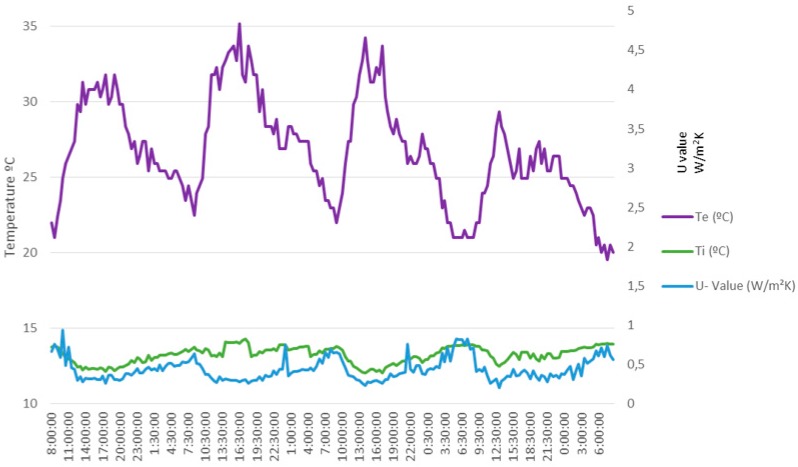
Measures taken with the heat flow metre after the energy retrofitting.

**Figure 12:**
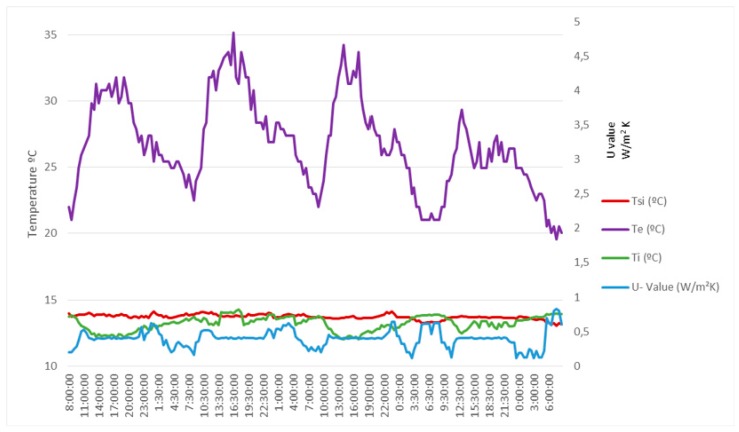
Measures taken with the developed *U-Value* metre after the energy retrofitting.

The energy retrofitting process consisted of an external insulation and finish system (EIFS). After it, the obtained *U-Values* by means of the assembly of Figure 9 are shown in Figure 11 and Figure 12. The first shows the heat flow metre measured *U-Value* and the obtained average *U-Value* is 0.43 W/(m^2^ K). The second shows the developed metre measured *U-Value* and the obtained average *U-Value* is 0.42 W/(m^2^ K). As can be verified, there is no practical difference between both metres (difference less than 2%). However, the very beneficial result of the energy retrofitting can be clearly observed. In fact, the *U-Value* has dropped more than 2 W/(m^2^ K).

Finally, it is important to point out that the data in Figure 10 was obtained in winter $\left(T_{i}>T_{e}\right)$, while those in Figure 11 and Figure 12 were obtained in summer $\left(T_{e}>T_{i}\right).$ Of course, while the test meets the standard ISO 9869-1:2014 [26], the time of year in which it is carried out should not influence, therefore the obtained result should be substantially the same.

## 5. Discussion

In new or under construction buildings with the knowledge that we have today of the construction materials as well as with the increasingly demanding regulations in the countries, the need to measure *U-Value* in the envelope of a building is generally one-off. However, all countries have huge building parks that were built with less demanding regulations (depending on their lifetime, perhaps with none). This huge body of existing buildings needs to be energy retrofitted to achieve levels of efficiency aligned with current requirements. It is precisely in this field, the one of energy retrofitting, where the *U-Value* knowledge is most needed and its measure must be carried out generally at particular dwellings level and even with several measures in each one.

When many measures of *U-Value* in buildings are needed in a short time, the methodology based on the direct measurement of the heat flow through the building envelope is impractical. Using this methodology, the only probable way to take many measures in few days is to work with as many devices at once as measures needed. This is unfeasible as well as very expensive.

The proposed methodology in this paper and the developed *U-Value* metre, which allows it to be carried out, resolves the problem. As we have shown in this paper and regarding the existing devices, our developed *U-Value* metre improves the current technology in the following aspects:

- It has a modular structure.
- It is made up of different modules configurable to customer needs.
- The module connection is extremely easy; no cable is needed; every connection is made wireless and in plug and play mode.
- It is capable of taking more than one measurement at a time; it is practically unlimited in this aspect. A *master + indoor* module can be managing tens or hundreds of *indoor* and *outdoor* modules.
- The developed *U-Value* metre and thanks to the *XBee + ZigBee* communication features allows for a communication range of more than 100 m in closed spaces and 1 km in open spaces. This provides the user to carry out measurements on all dwellings in one building or in several buildings simultaneously.
- The metre can decide automatically when a *U-Value* measurement is correct (the steady-state has been reached); thus the time spend is strictly the necessary, which allows shortening measurement times.
- Moreover, the user, based on our experience, can set the correct measurement time and the *U-values* gap in it.
- The developed *U-Value* metre can also work as usual, i.e., programmed for closed duration tests.
- The developed *U-Value* metre is provided with a friendly and ergonomic user interface.
- Independently of the installed modules, the developed *U-Value* metre acts as a single instrument; therefore it is able to manage all the measures at once and processed them as the user needs.
- The user interface and thanks to its VI can present the information in different ways as a data sheet or graphically.
- The devices present in the market process the data off-line, which further slows down the *U-Value* measure; the developed *U-Value* metre processes data in real time and provides the *U-Value* immediately.
- Measured data are saved in situ an also they can be sent to any part of the world via internet. In fact the user can govern the developed *U-Value* metre remotely.
- The internet connection of the developed *U-Value* metre can be made by way of cable, wi-fi, mobile phone network or even by modem radio.
- The mentioned features allow taking the necessary *U-Value* measurements in a large number of dwellings at once and in a short period of time. Today with the current devices available in the market the feature it is impossible.
- The developed *U-Value* metre is quite accurate. In fact, when it was compared with the current metre based on heat flow, the deviation has bwaseen always less than 2%. Not even this small deviation is a problem, since we make differential measures, i.e., we measure before and after the building energy retrofitting, and then we subtract the measures in order to get the obtained improvement.
- Regarding costs, the comparison between the developed *U-Value* metre and the current one based on heat flow is very favourable to the former. In fact, for one measurement point, the cost of the *master + indoor* module plus the *outdoor* one, including sensors in both, is around 240 €. Conversely, only the heat flow sensor costs more than 500 €. A commercial full instrumentation channel for one measurement point costs several thousand euros. If the user wants to carry out more than one measurement at a time, the cost increases a lot and the difference between one system and another can be several orders of magnitude.
- Despite the developed *U-Value* metre cost is cheaper than current existing devices it may be even smaller as it is only necessary to acquire the modules needed.

## 6. Conclusions

The thermal transmittance measurement (*U-Value*) is mandatory in building energy retrofitting, since by means of before and after measurements, the specialist can check the quality of the building energy retrofitting carried out. 

Today, the market offers *U-value* meters based on the heat flow measurement through the wall whose application to building energy retrofitting can be expensive and probably impractical; especially if many measurements are needed in a short time or even worse if many measurements must be made at once. 

This paper shows that from well-known physical laws, it is possible to deal the thermal transmittance measurement from different physical variables other than heat flow through the building envelope. Specifically a methodology based on the measurement of three temperatures has been described: wall outdoor surrounding, wall indoor surrounding and wall indoor surface. The measurement of these three temperatures can be made in a cheap and easy way; thus the practical application of this methodology can give rise to a new generation of *U-Value* metres.

Indeed, based on our methodology, this paper presents the development of a new *U-Value* metre. This was compared to the one currently used based on heat flow working together on a same test. The results show that the developed *U-Value* metre meets a series of features that make it ideal to be applied in building energy retrofitting. 

In summary, the developed *U-Value* metre offers a cheap, quick, reliable and simple way to measure *U-Values*. It is especially indicated when the amount of measures to be carried out is great and even more if these must be made at once.
